# Editorial: Computational models of brain in cognitive function and mental disorder

**DOI:** 10.3389/fpsyt.2023.1230587

**Published:** 2023-11-27

**Authors:** Rubin Wang, Jianzhong Su

**Affiliations:** ^1^School of Mathematics, Institute for Cognitive Neurodynamics, East China University of Science and Technology, Shanghai, China; ^2^Department of Mathematics, University of Texas at Arlington, Arlington, TX, United States

**Keywords:** computational models, neuroscience, cognitive function, mental disorder, neurodynamics

The computational models in neuroscience are to utilize the modern computational tools to mimic brain behavior and to provide insight for the inner working of cognitive functions including their abnormal states in mental disorder. These cognitive models work at different levels of neuronal activities, from subcellular neuronal networks to full brain dynamics, and neuronal activates across different spatial and temporal scales is interactional and often ovelapping. It is vital for understanding and testing hypothesis in these models to understand how brain functions at normal cognitive processes as well as brain's diseased states in mental disorder patients.

The goal of this computational modeling Research Topic is to set a forum to enhance communication for quantitative explorations of the inner working of the brain cognitive dynamics, in both normal and pathological cognitive states. The authors study integrated network models at a single level or multiple levels of the brain, develop models of specific brain function or behavior, and simulate the models to mimic mechanisms of mental disorder from theoretical and computational methods.

This Research Topic covers a full range of Research Topics including the analysis of experimental data from cognitive neuroscience, cognitive disorder, mental disorder as well as theoretical models in neurodynamics using tools from mathematics and physics, computer science, etc.

This Research Topic included eight research papers and one review paper. In “*Category learning in a recurrent neural network with reinforcement learning*” by Zhang et al., the authors constructed a deep reinforcement neural learning model by combining a recurrent neural network (RNN) with reinforcement learning. They illustrated the category learning process and discussed how the machine learning process is represented in the neuron network. In “*Individual prediction of hemispheric similarity of functional connectivity during normal aging*” by Zhang, the author calculated the hemispheric functional connectivity (HSFC) through Pearson correlation of brain signals, then the author further evaluated the variability of individual recognition of HSFC during the aging process. In “*Gender differential item functioning analysis in measuring computational thinking disposition among secondary school students”* by Sovey et al., the research assessed gender's effects on students' ability in using computational thinking and the evaluate quantities include cognitive, affective, and conative dispositions. In “*Brain network changes in adult victims of violence*” by Shymanskaya et al., the authors compared brain network changes among two groups: self-identified victims of violence and the control group of individuals who did not identify themselves as victims. Four large-scale brain networks, the default mode network, the salience network, the fronto-parietal network, and the dorsal attention network were included in this study. In “*Turing instability mechanism of short-memory formation in multilayer FitzHugh-Nagumo network*” by Wang and Shen, a theoretical study is presented. The authors analyzed pattern properties of a network of neurons modeled by FitzHugh-Nagumo model, a relative simple model of oscillatory neuronal activity. Particularly, the authors studied neural activity patterns on a multilayer network where the coupled neuronal system is random network. In “*Dissociated deficits of anticipated and experienced regret in at-risk suicidal individuals”* by Ai et al., the authors studied subclinical youth with suicidal ideation and compared their quantitative behavior with a control group of youth who do not have suicidal ideation. They studied research subjects' responses in regret anticipation and experience during a value-based decision-making process. In “*Data-driven evolutionary game models for the spread of fairness and cooperation in heterogeneous networks*” by Li et al., the authors built evolutionary game models based on the experimental phenomena and data. Through the research, they showed a joint effect of social preference and network heterogeneity on promoting prosocial behaviors. In “*The distribution and heterogeneity of excitability in focal epileptic network potentially contribute to the seizure propagation*”, by Fan et al., the authors used a connected network of neuronal units that have focal nodes prominently as their epilepsy model. Through computer simulation, they established a timescale difference that separated epileptic network model from non-seizure network. They concluded that factors affecting seizure occurrence are the connectivity patterns of focal network nodes and the network's ability to modulate the distribution of network excitability. Finally in a review paper “*Understanding mental health through computers: An introduction to computational psychiatry”*, by Martínez and Santamaría-García, the authors performed a literature review for the field of computational psychiatry. They indicated the computational models have been established as a new tool in the study of mental disorders and problems. They suggested modeling integration by models of different neuronal levels will create computational phenotypes that are highly valued in clinical study and neuroscience research. They articulated that modeling study can be valuable in assisting physicians in precision psychiatry and the field of computational psychiatry has a strong potential to continue to grow as a new branch of computational neuroscience.

## Author contributions

All authors contributed to manuscript revision, read, and approved the submitted version.

